# Supplementary material for: Self-filtering narrowband high performance organic photodetectors enabled by manipulating localized Frenkel exciton dissociation
Source: Nat Commun. 2020 Jun 8;11:2871. doi: 10.1038/s41467-020-16675-x (PMC7280211; doi:10.1038/s41467-020-16675-x)
Supplement: Supplementary file 1 — Supplementary Information [file 41467_2020_16675_MOESM1_ESM.docx]

**Self-filtering narrowband high performance organic photodetectors enabled** **by manipulating localized Frenkel exciton dissociation**

Xie et al.

**Supplementary Figures**


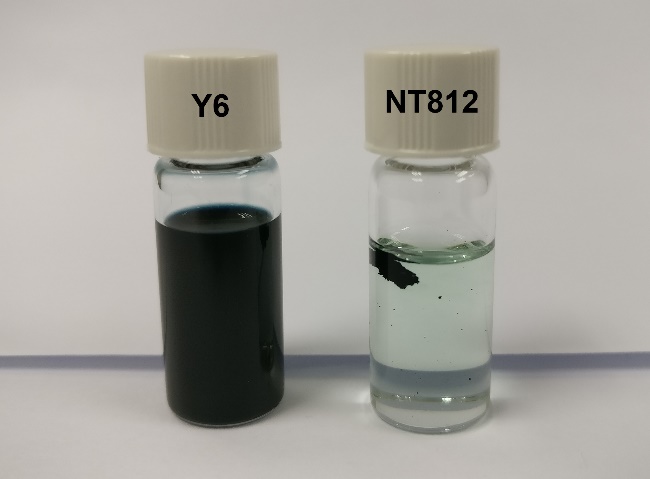


**Supplementary Figure 1| Appropriate solvent for sequential solution processing.**  Solubility of Y6 (15mg ml^−1^) and NT812 (2mg ml^−1^) in solvent CF.





**Supplementary Figure 2| ToF-SIMS fragment structure.** The structure of the secondary ion C_74_H_69_F_4_N_8_O_2_S_5_^−^ used to monitor the distribution of Y6.





**Supplementary Figure 3| Comparison of the SF-narrowband OPD with thick BHJ device.** EQE curves of our EDN OPD (SF-narrowband OPD) and reported CCN OPD (thick BHJ device) device with the same active-layer thickness (800nm) under −0.1 V bias.





**Supplementary Figure 4| Absorption characteristic of the donor material.** UV-vis absorption spectrum of thin film of NT812.





**Supplementary Figure 5| Transmittance spectrum characteristic.** The transmittance spectrum of glass/ITO/PEDOT:PSS(30 nm)/NT812(750 nm).

**

**

**Supplementary Figure 6| Characterization of self-filtering property.** Responsivity of the reference device (green solid line) and the SF-narrowband OPD with double donor layers (blue solid line) under −0.1 V bias; the curve of *S* (red dashed line) and the value of *T* at 860 nm.

**

**

**Supplementary Figure 7| Maintaining narrowband response at high bias voltage.** EQE curves of the SF-narrowband OPD with double donor layers under different voltage biases as indicated.


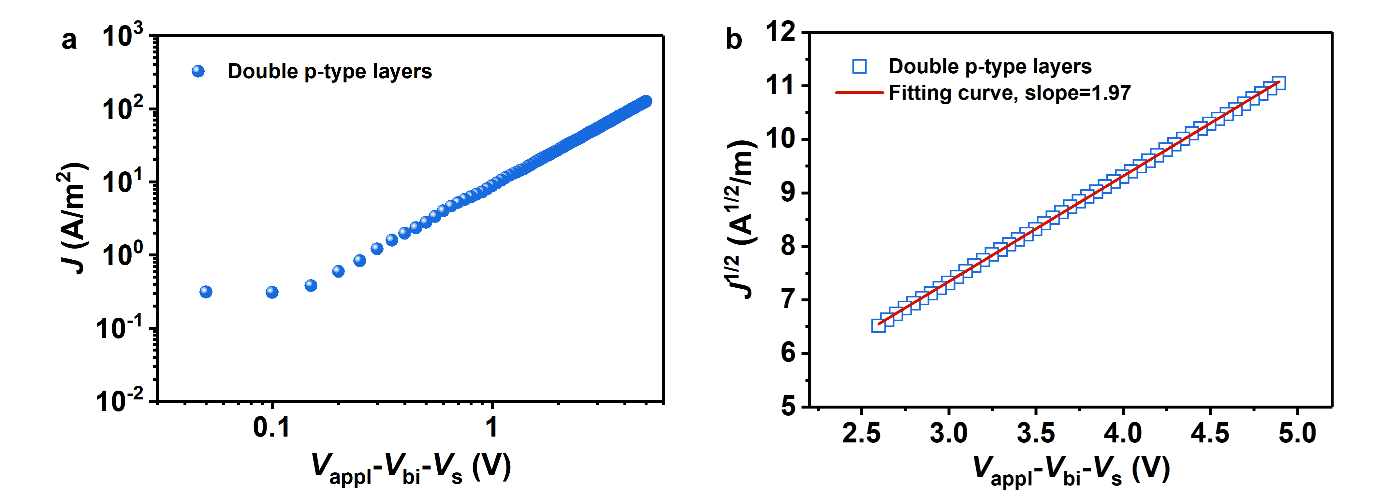


**Supplementary Figure 8| Hole mobility.** *J-V* curves (a) and *J*^1/2^-*V* curves (b) of hole-only device of the double donor layers with the device structure of ITO/PEDOT:PSS/ SF-HTL P3HT (150 nm)/donor front layer NT812 (750 nm)/MoO_3_/Ag.





**Supplementary Figure 9| Responsivity curve.** Responsivity of SF-narrowband OPD with double donor layers.





**Supplementary Figure 10| Logarithmic scaled specific detectivity curve.** Specific detectivity spectrum obtained from $J_{d}$ on a logarithmic scale.


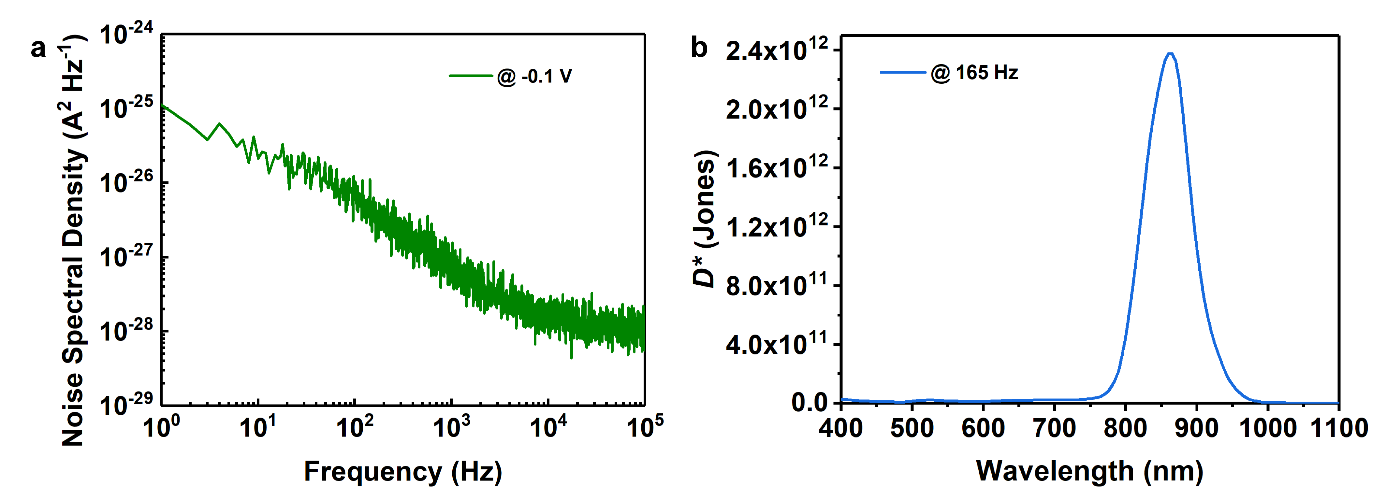


**Supplementary Figure 11| Specific detectivity curve calculated by noise current.** (a) Noise spectral density of SF-narrowband OPD with double donor layers (at −0.1 V bias). (b) Specific detectivity spectrum (at −0.1 V bias and 165 Hz) obtained from noise current.





**Supplementary Figure 12|** **Light intensity dependence of EQE.** The curve of EQE versus the light intensity at 850 nm under −0.1 V bias.


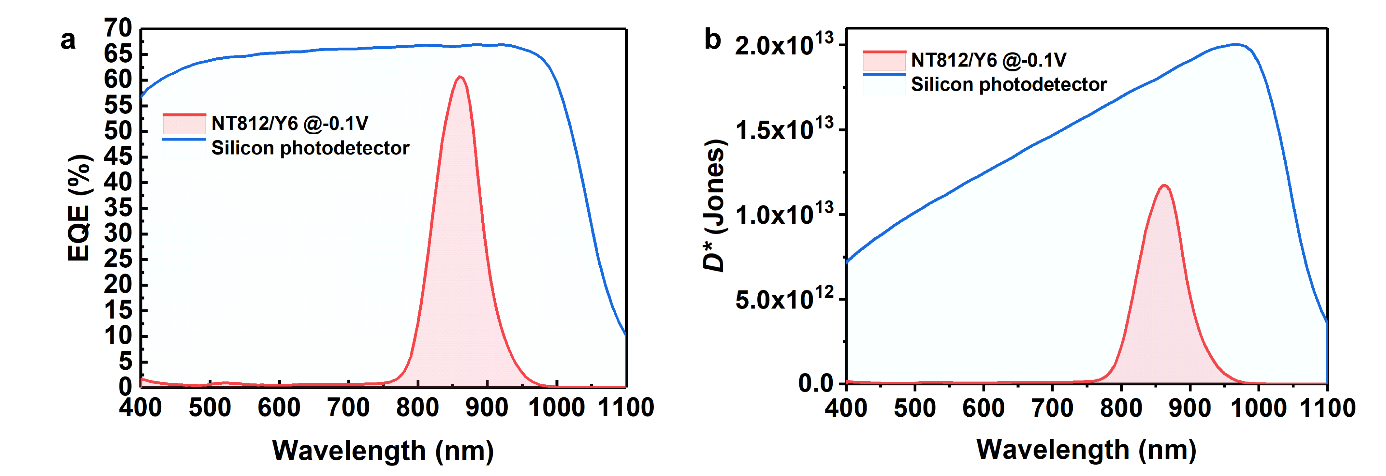


**Supplementary Figure 13|** **Comparable detection performance.** (a) EQE spectra and (b) Specific detectivity spectra of the SF-narrowband OPD and commercialized silicon photodetectors


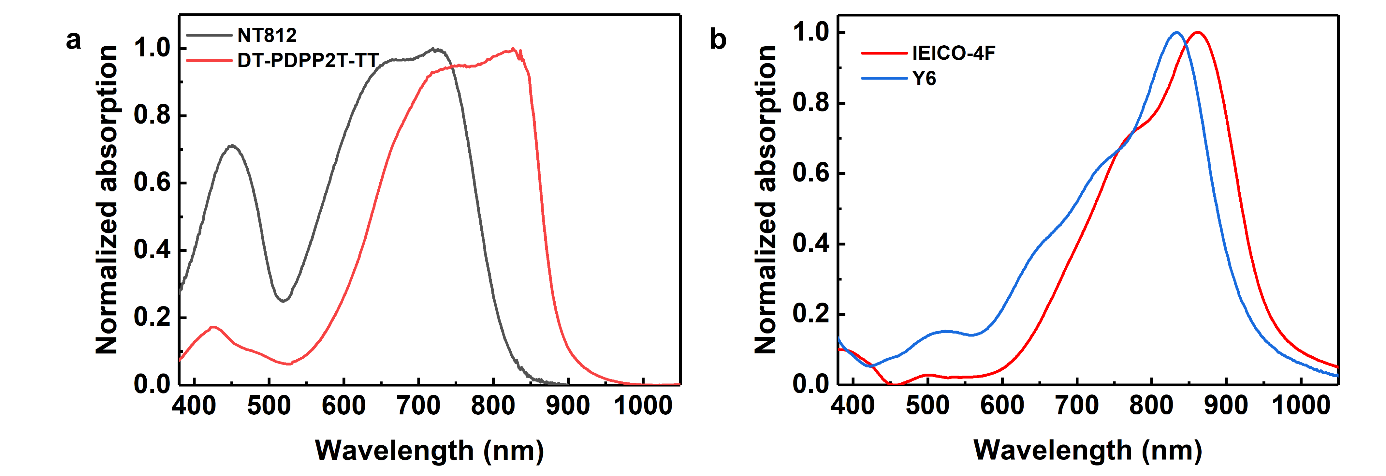


**Supplementary Figure 14|** **Universality of self-filtering narrowband OPDs.** Normalized UV-Vis absorption spectra of (a) donor materials and (b) acceptor materials.


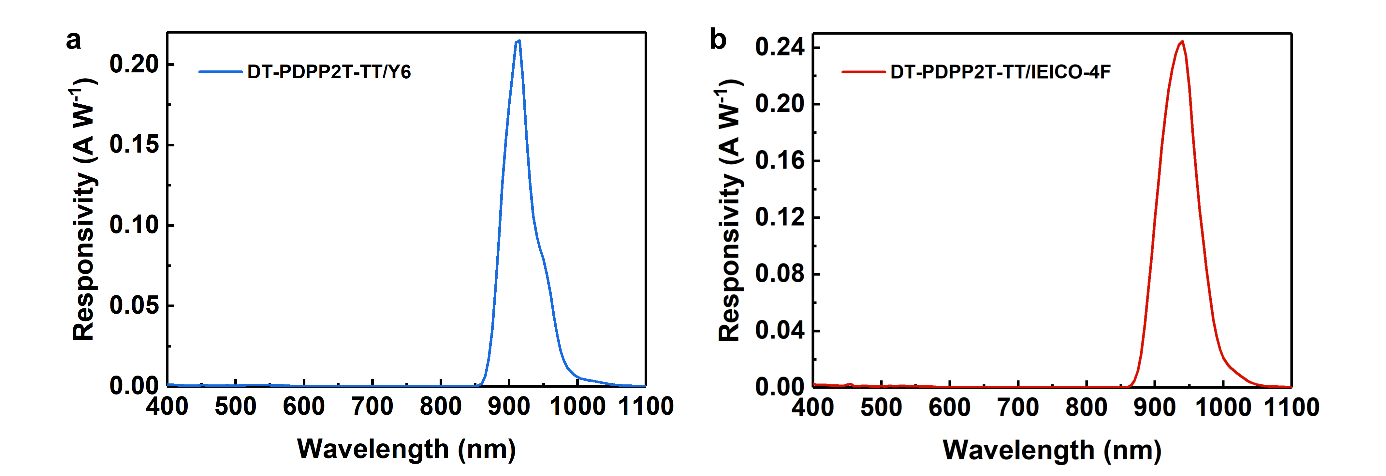


**Supplementary Figure 15| Responsivity of self-filtering narrowband OPDs.** Responsivity of SF-narrowband OPDs based on (a) DT-PDPP2T-TT/Y6 and (b) DT-PDPP2T-TT/IEICO-4F. Both devices were tested under −0.1 V bias.

**Supplementary Notes**

**Supplementary Note 1. Monte Carlo Simulation to extract the exciton diffusion length.**

We calculated the exciton diffusion coefficient $D$ of the front-layer material NT812 using the Monte Carlo simulation method proposed by Mikhnenko *et al.* Measuring and modeling photoluminescence (PL) decays of semiconductor blends with exciton quenching molecules can result in extracting exciton diffusion parameters in organic semiconductors. PCBM is used as exciton quencher typically. Excitons are crated in the semiconductor diffuse toward exciton quenching molecules, where they undergo dissociation (quenching). As a result, the PL decay time of a semiconductor:PCBM blend is shorter than that of pristine semiconductor. PL decay times of samples of various PCBM fractions can be compared using the relative quenching efficiency $Q$ that is defined as:

$Q=1-\frac{\int{PL}_{\mathrm{blend}}dt}{\int{PL}_{\mathrm{pristine}}dt}$ (1)

where blend ${PL}_{\mathrm{blend}}$ and pristine ${PL}_{\mathrm{pristine}}$ are normalized to the value at time zero PL decays of a polymer semiconductor:PCBM blend and pristine polymer, respectively. The relative quenching efficiency $Q$ depends on the PCBM concentration, blend morphology and exciton diffusion coefficient. If the former two parameters are known, then the relative quenching efficiency bears information about exciton diffusion coefficient. In this study, a cubic simulation box with the edge length of 50 nm and periodic boundary conditions was considered to be a continuous medium of polymer phase, in which PCBM quenchers were placed. PCBM molecules were approximated as balls of 1 nm in diameter. Two types of morphologies were considered, including intimate mixture and phase separated PCBM clusters of a certain size. The intimate mixture was modeled by randomly placing PCBM molecules into the simulation box. As shown in Fig. 2a, the pristine film of NT812 showed deconvoluted mono-exponential PL decay time of 906.3 ps, and PL decay time of NT812:PCBM blend with PCBM volume fraction of 0.05% was 722.4 ps, according to the Supplementary Equation (1), the relative quenching efficiency of this blend was: $Q=1-\frac{722.4}{906.3}=0.203$. The density of NT812 was set as 1.3 g cm^−3^. We input these parameters into the Monte Carlo Simulation program and resulted in the initial diffusion coefficient $D$ of 19.5 × 10^4^  cm^2^ s^−1^, the diffusion length $L$ was then calculated using the relation of $L=\sqrt{D\tau}$ as 13.3 nm.

**Supplementary Note 2. Hole mobility the double donor layers.** In order to demonstrate photoinduced holes can travel through the thick double donor layers efficiently, we evaluated the carrier mobility of the double donor layers using the space-charge limited current (SCLC) method with a device structure of ITO/PEDOT:PSS/ SF-HTL P3HT (150 nm)/donor front layer NT812 (750 nm)/MoO_3_/Ag. The hole mobility was determined by fitting the dark current to the model of single carrier SCLC, which is described by the equation:

$J=\frac{9}{8}\varepsilon_{0}\varepsilon_{r}\mu\frac{V^{2}}{d^{3}}$ (2)

where $J$ is the current, $\mu$ is the zero-filed mobility, and $\varepsilon_{0}$= 8.85×10^−12^ F/m and $\varepsilon_{r}$=3 are the permittivity of free space and relative permittivity of the material, respectively. *V* is the effective voltage, and *d* is the thickness of whole double donor layers, which was set at 921 nm, as measured by the profilometer. The effective voltage can be obtained by subtracting the built-in voltage (*V*_bi_) and the voltage drop (*V*_s_) from the substrate’s series resistance from the applied voltage (*V*_appl_), *V* = *V*_appl_-*V*_bi_-*V*_s_. The resulted *J-V* cure is shown in Supplementary Fig. 8a. The hole mobility was then evaluated from the slope of the *J*^1/2^-*V* curve (1.97, as shown in Supplementary Fig. 8b) as 1.02 × 10^−3^ cm^2^ V^−1^ s^−1^.

**Supplementary Note 3. Calculated thermal noise and detectivity.** The magnitude of thermal noise^1^ ($i_{\mathrm{th}}$) is expressed as Supplementary Equation (3):

$i_{th}=\sqrt{\frac{4kTB}{R_{L}}}$ (3)

where $k$ is the Boltzmann constant, $T$ is temperature in Kelvin, which was 293 K under the experimental condition, $B$ is the noise measurement bandwidth, which was set as 165 Hz, and $R_{L}$ is the shunt resistance in the device, which was 480 $M\Omega$ obtained from the *I-V* slope.

The magnitude of the dark current noise ($i_{d}$) is expressed as Supplementary Equation (4):

$i_{d}=\sqrt{2qI_{d}B}$ (4)

where $q$ is the electron charge, $I_{d}$ is the dark current, which was 2.08 × 10^−10^ A at −0.1 V bias. So the calculated noise current can be expressed as Supplementary Equation (5):

$i_{\mathrm{cal}}=\sqrt{i_{d}^{2}+i_{\mathrm{th}}^{2}}=1.29\times{10}^{-13}A$ (5)

And the calculated detectivity can be can be expressed as Supplementary Equation (6):

$D^{*}= \frac{R\sqrt{AB}}{i_{\mathrm{cal}}}$ (6)

Where $R$ is the responsivity and $A$ is the device area. The calculated detectivity demonstrated a peak value of 9.5 × 10^12^ Jones at 860 nm, which was only slightly lower than that obtained without the thermal noise of 1.2 × 10^13^ Jones.

**Supplementary References**

1. Yang, D. & Ma, D. Development of organic semiconductor photodetectors: from mechanism to applications. *Adv. Optical Mater*. **7**, 1800522 (2019).
